# The incidence of and risk factors for radiation pneumonitis in patients treated with simultaneous bevacizumab and thoracic radiotherapy

**DOI:** 10.1186/s13014-024-02458-x

**Published:** 2024-05-30

**Authors:** Feihu Chen, Jiling Niu, Min Wang, Hui Zhu, Zhijun Guo

**Affiliations:** 1 Department of Radiation Oncology, Shandong Cancer Hospital and Institute, Shandong First Medical University, Shandong Academy of Medical Sciences, Jinan, China; 2 Department of Intensive Care Unit, Shandong Cancer Hospital and Institute, Shandong First Medical University, Shandong Academy of Medical Sciences, 440 Jiyan Road, Jinan, 250117 Shandong China

**Keywords:** Radiation pneumonitis, Bevacizumab, Thoracic radiotherapy, Non-small cell lung cancer

## Abstract

**Background:**

First-line chemotherapy combined with bevacizumab is one of the standard treatment modes for patients with advanced non-small cell lung cancer (NSCLC). Thoracic radiotherapy (TRT) can provide significant local control and survival benefits to patients during the treatment of advanced NSCLC. However, the safety of adding TRT has always been controversial, especially because of the occurrence of radiation pneumonia (RP) during bevacizumab treatment. Therefore, in this study, we used an expanded sample size to evaluate the incidence of RP when using bevacizumab in combination with TRT.

**Patients and methods:**

Using an institutional query system, all medical records of patients with NSCLC who received TRT during first-line chemotherapy combined with bevacizumab from 2017 to 2020 at Shandong Cancer Hospital and Institute were reviewed. RP was diagnosed via computed tomography and was classified according to the RTOG toxicity scoring system. The risk factors for RP were identified using univariate and multivariate analyses. The Kaplan–Meier method was used to calculate progression-free survival (PFS) and overall survival (OS).

**Results:**

Ultimately, 119 patients were included. Thirty-eight (31.9%) patients developed Grade ≥ 2 RP, of whom 27 (68.1%) had Grade 2 RP and 11 (9.2%) had Grade 3 RP. No patients developed Grade 4 or 5 RP. The median time for RP occurrence was 2.7 months (range 1.2–5.4 months). In univariate analysis, male, age, KPS score, V_20_ > 16.9%, V_5_ > 33.6%, PTV (planning target volume)-dose > 57.2 Gy, and PTV-volume > 183.85 cm^3^ were correlated with the occurrence of RP. In multivariate analysis, male, V_20_ > 16.9%, and PTV-volume > 183.85 cm^3^ were identified as independent predictors of RP occurrence. The mPFS of all patients was 14.27 (95% CI, 13.1–16.1) months. The one-year and two-year PFS rates were 64.9% and 20.1%, respectively. The mOS of all patients was 37.09 (95% CI, 33.8–42.0) months. The one-year survival rate of all patients was 95%, and the two-year survival rate was 71.4%.

**Conclusions:**

The incidence of Grade ≥ 2 RP in NSCLC patients who received both bevacizumab and TRT was 31.9%. Restricting factors such as V_20_ and PTV will help reduce the risk of RP in these patients. For patients who receive both bevacizumab and TRT, caution should be exercised when increasing TRT, and treatment strategies should be optimized to reduce the incidence of RP.

## Background

First-line chemotherapy combined with bevacizumab is one of the standard treatment modes for patients with advanced non-small cell lung cancer (NSCLC) [1, 2]. The efficacy of bevacizumab in extending OS and progression-free survival (PFS) when added to platinum doublet chemotherapy as the first-line treatment for advanced NSCLC has been proven. In ECOG4599, the addition of bevacizumab to paclitaxel–carboplatin (PC) prolonged overall survival (OS) by two months compared with chemotherapy alone (median OS, 12.3 vs. 10.3 months; *p* = 0.003) [1]. For Chinese patients in the BEYOND trial, OS was prolonged in patients who received bevacizumab plus chemotherapy (median OS, 24.3 vs. 17.7 months; *p* = 0.015) [2]. During the maintenance phase, ECOG5508 [3] reported that the combined pemetrexed and bevacizumab maintenance group had a better objective response rate (ORR) than the single-agent maintenance group. Similar results were also obtained in the PRONOUNCE and COMPASS studies [4, 5].

Most advanced lung cancer patients will incorporate thoracic radiotherapy (TRT) during treatment to achieve a higher local response rate. Previous literature has reported that TRT can significantly prolong the survival of patients whether it is used as a salvage or consolidation treatment method [6]. The American Society for Radiation Oncology evidence-based clinical practice guidelines have confirmed that TRT, as a salvage treatment measure, has significant survival differences when added during disease progression. Subsequent studies have also demonstrated the value and status of TRT as a salvage treatment [7–10]. Additionally, TRT as a consolidating treatment method should not be ignored. A meta-analysis involving 757 advanced NSCLC patients with distant metastasis showed a median 5-year OS rate in the consolidated TRT group of 29%, much higher than 2% in the population who did not undergo consolidation TRT [11]. Similarly, adding consolidation radiotherapy to maintenance chemotherapy significantly benefited PFS (9.7 m vs. 3.5 m, *p* = 0.01) [12]. In addition, multiple studies have confirmed that whether the first-line treatment is chemotherapy, EGFR-TKI, or immunotherapy, using TRT as consolidation radiotherapy can significantly prolong the survival of patients [13–15].

However, the risk factors for RP in patients treated with simultaneous bevacizumab and TRT have always been a concern for clinical doctors [16, 17]. Six patients receiving radiotherapy combined with bevacizumab were included in a small phase I clinical trial. Of these, 5 patients developed RP, and 2 had RP higher than Grade 3; the incidence of RP was 66.7% [18]. In addition, the combination of bevacizumab and TRT significantly increased the incidence of other adverse reactions, such as bleeding and tracheoesophageal fistula (TEF). Given the high toxicity of bevacizumab combined with TRT reported in multiple small-sample studies, no further studies have combined the two methods. However, the previous studies had no statistical significance due to the small sample size. Nevertheless, the combination of bevacizumab and TRT should not be dismissed because of the significant survival benefits brought to patients. Therefore, in this study, we used an expanded sample size to evaluate the incidence of RP when using bevacizumab in combination with TRT.

## Patients and Methods

### Study design and patients

This study aimed to assess the incidence of RP and identify risk factors for patients treated with simultaneous bevacizumab and TRT. The medical records of all included patients between 2017 and 2020 at Shandong Cancer Hospital and Institute were reviewed using an institutional query system. Information such as gender, age, and radiation parameters were collected.

According to the 2017 lung cancer diagnosis and treatment guidelines, the inclusion criteria were as follows: patients with conclusive imaging evidence and pathological results that confirmed the diagnosis of stage IV lung adenocarcinoma that was driver gene negative, and patients who received TRT combined with bevacizumab during first-line chemotherapy. The tumour stage was assessed by systemic imaging (contrast-enhanced computed tomography [CT] of the chest and abdomen, bone scan, or positron emission tomography/computed tomography [PET-CT]) and brain imaging (either contrast-enhanced CT or magnetic resonance imaging [MRI]). Patients who had a history of ILD or poor essential lung function were excluded.

### Treatment protocol

All patients underwent intensity-modulated radiotherapy and 3-dimensional conformal radiation therapy with photon therapy. V_5_ and V_20_ were recorded on each patient’s DVH chart of the radiotherapy plan. Total lung V_5_ and V_20_ are the percentages of total lung volume receiving radiation exceeding 5 Gy and 20 Gy, respectively. Bevacizumab, pemetrexed, and platinum were administered intravenously every three weeks at 15 mg/kg, 500 mg/m^2^, and 75 mg/m^2^, respectively. The dose of paclitaxel was 175 mg/m^2^. Data were collected from the medical records. The study was approved by the Ethics Committee of Shandong Cancer Hospital and Institute, performed in accordance with the Declaration of Helsinki and conducted under the supervision of Shandong Cancer Hospital and Institute.

### Diagnosis and classification of radiation pneumonitis

RP was diagnosed via CT. At least two senior radiologists independently evaluated the follow-up CT examination, and differences were resolved by consulting with a third senior radiologist. According to the patient’s clinical symptoms, laboratory test results, and treatment results, RP was scored from 1 to 5 points according to the RTOG toxicity scoring systems: Grade 0, no change; Grade 1, mild symptoms of dry cough or dyspnoea on exertion; Grade 2, persistent cough requiring narcotic or antitussive agents or dyspnoea with minimal effort but not at rest; Grade 3, severe cough unresponsive to narcotic antitussive agent or dyspnoea at rest, clinical or radiological evidence of acute pneumonitis, need for intermittent oxygen or steroids; Grade 4, severe respiratory insufficiency, need for continuous oxygen or assisted ventilation; and Grade 5, death caused by toxicity.

### Statistical analysis

All statistical analyses were performed using GraphPad Prism software v.8.0 (GraphPad, Inc., CA, USA) and SPSS statistical software v.26 (IBM Corp., NY, USA). The Kaplan‒Meier method was used to calculate PFS and OS. PFS was defined as the time from treatment commencement of bevacizumab and TRT to confirmed disease progression or death of any cause. Overall survival (OS) was defined as the period from the date from treatment commencement of bevacizumab and TRT to the date of death.

The continuous variables obtained from statistics were converted into categorical variables. Univariate and multivariate analyses using binary logistic regression were conducted to analyse the risk factors for RP. Meanwhile, for noncategorical variables, receiver operating characteristic (ROC) curves were first generated to determine the optimal cut-of value. The cut-of value was determined according to the maximum Youden index after considering sensitivity and specificity. Then, the continuous variables were converted to categorical variables. Two-sided p values < 0.05 were considered statistically significant. Significant variables in the univariate analysis (i.e., those with a p value of ≤ 0.05) were included in the multivariate analysis. For variables with collinearity, those with a smaller p value and that were representative were chosen for multivariate analysis. When calculating patient baseline characteristics, based on the limited exposure of radiation therapy to organs at risk, we grouped total lung V_20_ at 25% and V_5_ at 60%. The number of cycles of bevacizumab before TRT was divided into groups with a cutoff of four cycles because the guidelines recommend that bevacizumab should be used with chemotherapy for four cycles and then maintained alone.

## Results

### Patient characteristics

We screened 425 patients treated with first-line chemotherapy combined with bevacizumab, 119 of which underwent TRT and were included in our study analysis. The clinical and radiation dosimetry characteristics of all patients are shown in Table 1. The median age was 65 years (range 42–90 years), and most patients were male (83.2%). The median follow-up time was 41.4 months. The median PTV (planning target volume)-dose was 60 Gy (range 45–75 Gy). Most patients had PTV-dose concentrated between 50 and 60 Gy (*n* = 103, 86.6%). Ten patients received doses of more than 60 Gy. V_20_ was well constrained (median 13.2%; range 8.2–33.3%), but only 11 individuals exceeded the 25% V_20_ limit. The radiotherapy mode for most patients was a fractionated dose of 2 Gy per session, with 30 exposures. However, five patients chose a single 8 Gy hypofractionated radiotherapy treatment; the final total radiation amount was 48–56 Gy. Regarding the timing of adding TRT, TRT was performed on 13 patients (10.9%) who developed primary progressive disease (PD) during bevacizumab treatment. TRT consolidated the primary lesions in the remaining patients without PD. Finally, more than half of the patients received TRT before the first four cycles of bevacizumab use (*n* = 93, 78.2%) (Table 1).

**Table 1:** Baseline characteristics of patients

Characteristics	Total (%) (n = 119)
Gender	
Male	99 (83.2)
Female	20 (16.8)
Age (y)	
(Median, range)	65(42–90)
< 65	69 (58)
≥ 65	50 (42)
Smoking history	
No	69 (58)
Yes	50 (42)
KPS score	
< 80	4 (3.4)
≥ 80	115 (96.6)
PTV-dose (Gy)	
(Median, range)	60 (45–75)
< 50	6 (5.0)
50–60	103 (86.6)
> 60	10 (8.4)
Fractiondose (Gy)	
(Median, range)	2 (1.5-8)
> 3	14 (11.8)
≤ 3	105 (88.2)
Number of cycles of bevacizumab before radiotherapy	
≤ 4	26 (21.8)
> 4	93 (78.2)
PTV-volume (cm³)	
(Median, range)	183.85(20.2-950.4)
≤ 183.85	74 (62.2)
> 183.85	45 (37.8)
V_20_ total (%)	
(Median, range)	13.2 (8.2–33.3)
≤ 25	108 (90.8)
> 25	11 (9.2)
V_5_ total (%)	
(Median, range)	29.4 (10.4–86.7)
≤ 60	114 (95.8)
> 60	5 (4.2)
Timing of radiotherapy addition	
Consolidation	106 (89.1)
Salvage	13 (10.9)
Chemotherapy regimen	
PP	111 (93.3)
TP	8 (6.7)

Abbreviations: KPS: Karnofsky performance status; RP : radiation pneumonitis; PP: pemetrexed + carboplatin/cisplatin; TP: paclitaxel + carboplatin/cisplatin

### Incidence of RP

**Fig. 1:**
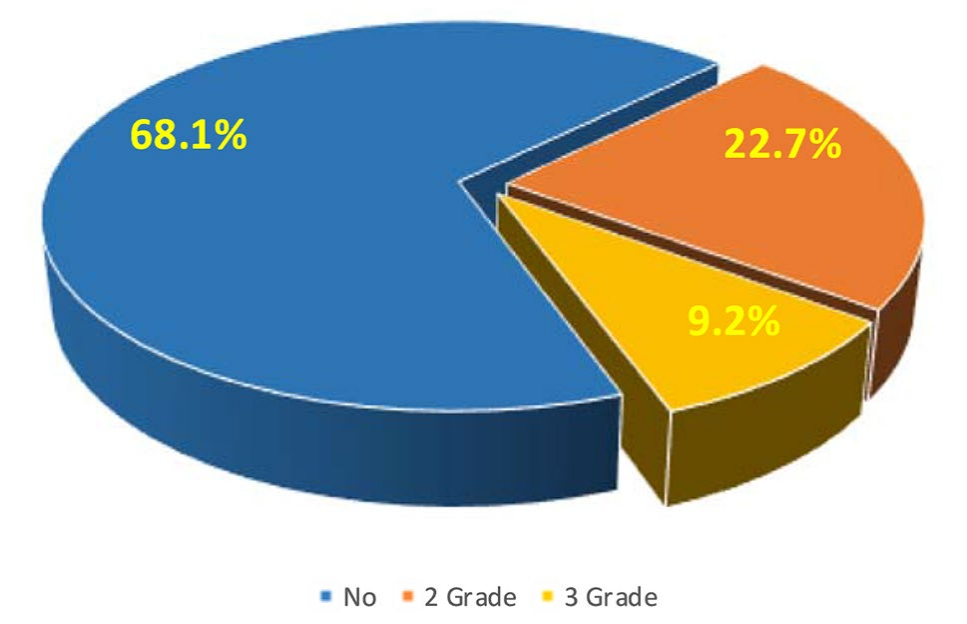
The incidence of ≥ 2 grade RP in 119 patients. There are 38 (31.9%) patients had developed ≥ 2 grade RP in total 119 patients, of which 27 (22.7%) had 2 grade RP and 11 (9.2%) had 3 grade radiation pneumonia. No one has developed 4 or 5 grade RP

**Fig. 2:**
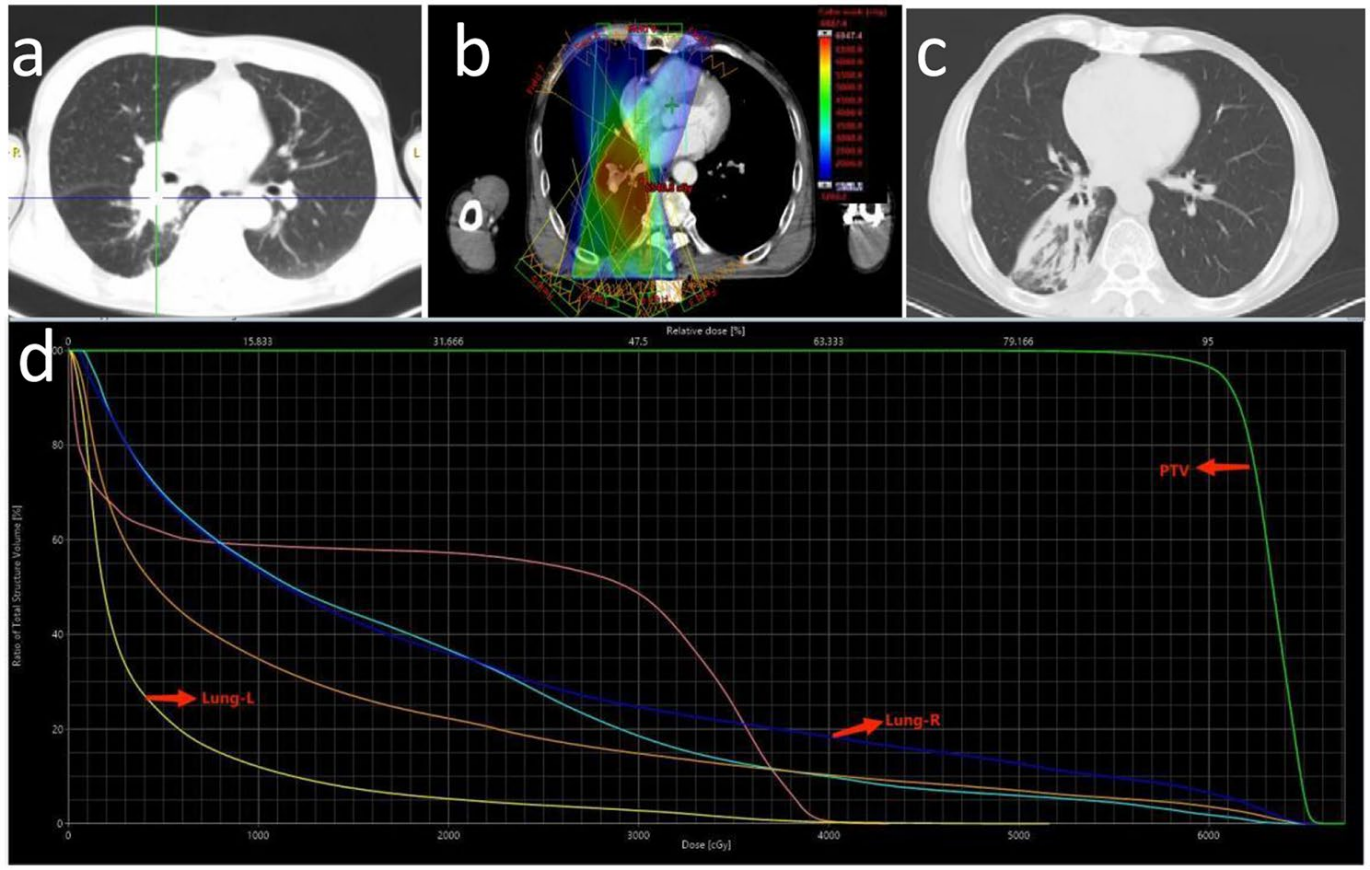
Representative images of one patient who experienced grade 3 RP after TRT. (**a**). Primary lesion. (**b**). Isodose curve of the treatment plan. (**c**) Three months after radiotherapy. (**d**) Dose distribution histogram of the total lung, right lung, PTV and left lung

**Fig. 3:**
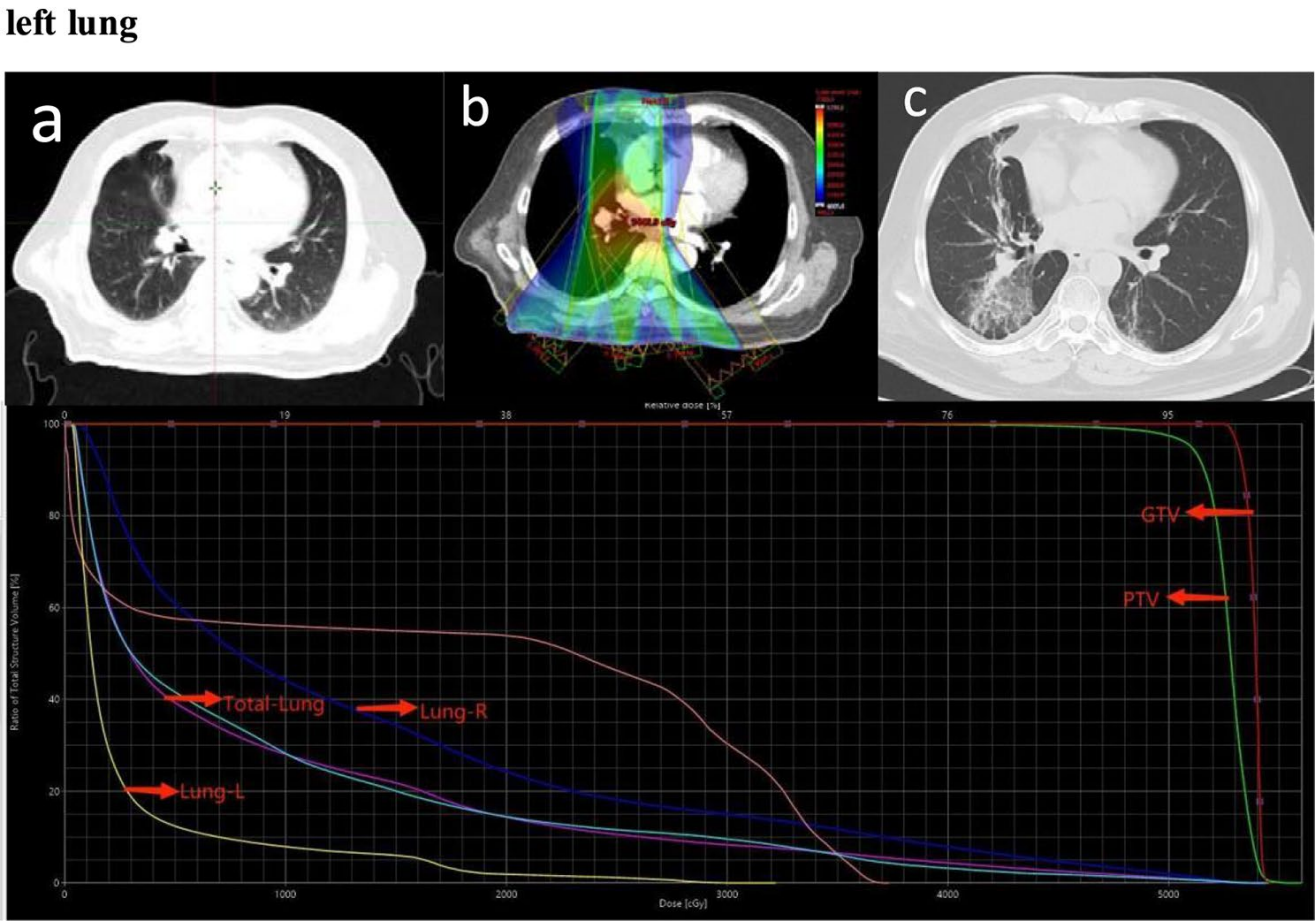
Representative images of one patient who experienced grade 3 RP after TRT. (**a**). Primary lesion. (**b**). Isodose curve of the treatment plan. (**c**) Three months after radiotherapy. (**d**) Dose distribution histogram of the total lung, right lung, left lung, PTV, GTV

Of 119 patients, 38 (31.9%) developed Grade 2 or higher RP, 11 (9.2%) developed Grade 3, and nobody developed Grade 4 or higher. The median time to RP after TRT was 2.7 months (range 1.2–5.4 months) (Fig. 1). Among the 38 patients who developed RP, 5 developed RP within one month after TRT, and one patient developed symptoms such as chest tightness and suffocation ten days after TRT and was diagnosed through CT. These five patients received a total dose of 60 Gy of TRT, with V_20_ values of 31.9%, 32%, 27%, 16.2%, and 24.3%. The patient with 31.9% V_20_ eventually developed Grade 3 RP. Two typical images from 11 cases of Grade 3 RP are shown in Figs. 2 and 3.

**Fig. 4:**
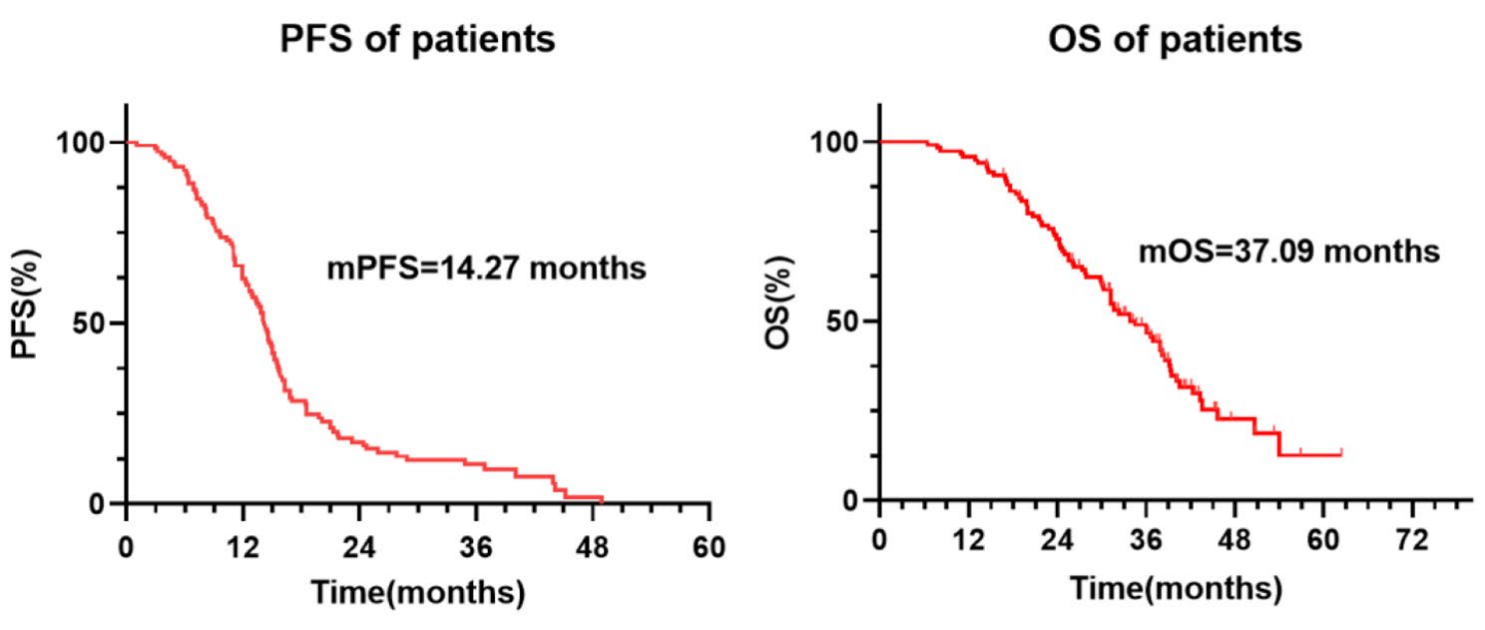
Survival analysis of patients receiving bevacizumab and TRT. All the patients’ mPFS was 14.27 months, and the mOS was 37.09 months

### Other adverse events

One of the patients developed a tracheoesophageal fistula (TEF) due to TRT, resulting in massive haemoptysis. The patient received a radiation dose of 54 Gy. V_20_ was well limited to 16.2%. Rescue measures such as haemostasis and tracheal intubation were urgently undertaken; unfortunately, the patient ultimately died. After discussion with the multidisciplinary cancer committee, it was agreed that the patient was susceptible to bevacizumab and radiotherapy, and the tumour invaded the trachea, resulting in a TEF.

## Risk factors for RP

### Univariate analysis

The optimal thresholds for PTV-volume, V_20_, V_5_, and PTV-dose based on the maximum Youden index are 183.85cm^3^, 16.9%, 33.6%, and 57.2 Gy, respectively. As shown in Table 2, as the lung radiation dosage increased, the overall risk of Grade ≥ 2 RP also increased. PTV-dose > 57.2 Gy (OR 9.56; 95% CI 3.86–23.68; *p* = 0.033), PTV-volume > 183.85cm^3^ (OR 1.01; 95% CI 1.00-1.02; *p* = 0.027), and total lung V_5_ > 33.6% (OR 1.10; 95% CI 1.07–1.14; *p* = 0.036) and V_20_ > 16.9% (OR 1.17; 95% CI 1.09–1.25; *p* = 0.022) were significantly correlated with RP. In addition to lung radiation dosimetry parameters, other clinical features were also included in the univariate analysis, and age (OR 2.63; 95% CI 1.22–5.66; *p* = 0.013), male (OR 4.01; 95% CI 1.10-14.56; *p* = 0.035), and KPS score (OR 2.32; 95% CI 1.07–5.06; *p* = 0.034) were also related to the occurrence of RP. History of smoking, fraction dose, and number of cycles of bevacizumab before TRT were unrelated to RP.

**Table 2:** Univariate and multivariate analysis of risk of developing RP

Risk Factors	Univariate analyses		Multivariate analyses	
	OR (95%CI)	p-value	OR (95%CI)	p-value
Gender				
Male				
Female	4.01(1.10-14.56)	**0.035**	4.32 (1.02–18.60)	**0.047**
Age				
≥ 65				
< 65	2.63 (1.22–5.66)	**0.013**	2.26 (0.84–6.04)	0.105
KPS score				
< 80				
≥ 80	2.32 (1.07–5.06)	**0.034**	1.81 (0.66–4.92)	0.248
Drinking history				
Yes				
No	0.84 (0.38–1.88)	0.669	NA	—
Smoking history				
Yes				
No	1.66 (1.37–1.71)	0.567	NA	—
Fraction dose (Gy)				
> 3				
≤ 3	1.06 (1.01–1.23)	0.423	NA	—
PTV-dose (Gy)				
> 57.2				
≤ 57.2	9.56(3.86–23.68)	**0.033**	NA	—
V_20_-total (%)				
> 16.9				
< 16.9	1.35 (1.31–1.52)	**0.012**	1.44 (1.37–1.51)	**0.029**
V_5_-total (%)				
> 33.6				
≤ 33.6	1.10 (1.07–1.14)	**0.036**	NA	—
PTV-volume (cm³)				
> 183.85				
≤ 183.85	1.01 (1.00-1.02)	**0.027**	1.004 (1.00-1.07)	**0.031**
Number of cycles of bevacizumab before radiotherapy				
≤ 4				
> 4	0.99 (0.95–1.04)	0.918	NA	—

Values in bold are statistically significant

Abbreviations: KPS: Karnofsky performance status; NA: Not applicable; OR: Odds ratio; RP : radiation pneumonitis; PTV: plan tumor volume

### Multivariate analysis

Subsequently, the individual factors with a correlation were included in the multivariate analysis. Because of the collinearity and strong correlation of lung radiation dose parameters, the ipsilateral lung V_20_ was converted as a categorical variable, and the variable with the smallest p value was included in the multivariate analysis. The results showed that male (OR 4.32; 95% CI 1.02–18.60; *p* = 0.047), PTV-volume > 183.85 Gy (OR 1.004; 95% CI 1.00-1.07; *p* = 0.031), and V_20_ > 16.9% (OR 1.44; 95% CI 1.37–1.51; *p* = 0.029) were independent predictors of RP. Therefore, strict restrictions on V_20_ and minimizing tumour volume before TRT may help reduce the risk of developing RP.

### Survival analysis

The median follow-up time was 41.4 months. At the time of the last follow-up, eight patients were lost; out of 119 patients, six did not experience PD. Among them, five patients were in the maintenance stage of bevacizumab, and only one was in the maintenance stage of pemetrexed and bevacizumab. The longest time without PD was 38.2 months, and the shortest was 14.4 months. In addition, 38 patients experienced PD and underwent second- or multiple-line treatment, with no deaths. The remaining patients eventually died after PD. The mPFS of all patients was 14.27 (95% CI, 13.1–16.1) months. Additionally, the one-year and two-year PFS rates were 64.9% and 20.1%, respectively. The mOS of all patients was 37.09 (95% CI, 33.8–42.0) months. The one-year survival rate of all patients was 95%, and the two-year survival rate was 71.4% (Fig. 4).

## Discussion

We conducted this retrospective study based on the high incidence (66.7%) of adverse reactions, mainly RP, when using bevacizumab in combination with TRT, as reported in previous small-sample studies [18]. We enrolled a total of 119 patients who underwent TRT during bevacizumab treatment, compensating for the insufficient sample size in previous studies. Our research results shows that the incidence of RP of grade 2 and above is 31.9% and the incidence of grade 3 RP is 9.2%. Fortunately, RP of grade 4 or above did not occur, thus our results did not exceed clinically acceptable limits. Subsequently, V_20_ > 16.9% and PTV-volume > 183.85 cm^3^ were identified as independent predictors of RP occurrence. In terms of survival, the combination of bevacizumab and TRT in our study resulted in significant survival benefits (mPFS, 14.27 months; mOS, 37.09 months). In summary, there was no severe RP of grade 4 or above, and the addition of TRT brought significant survival benefits to patients, simultaneous bevacizumab and TRT is feasible.

Previous studies have reported the incidence of RP in various treatment modes combination with TRT [19]. Under the traditional curative radiotherapy and chemotherapy mode, the incidence of RP is 13 -37% [20]. In addition, a systematic literature review and meta-analysis reported that the incidence of grade 3 and above RP ranged from 3.62 to 7.85% [21]. When immunotherapy is combined with TRT, the incidence of RP varies depending on the medication used. The incidence of grade 2 or above RP with programmed cell death ligand-1/ programmed cell death-1 (PD-L1/PD-1) inhibitors is 33.9%/15.1 -21.6%, and the incidence of grade 3 or above RP is 2.6 -3.3%/5.9 -11.7% [22–27]. For patients with positive driver genes, the incidence of grade 2 or above RP in patients receiving first generation epidermal growth factor receptor tyrosine kinase inhibitor (EGFR-TKI) combined with TRT is 44.78%, and the incidence of grade 3 or above RP is 8.96 -10% [28, 29]. When Osimertinib combined with TRT, the incidence of RP above grade 2 is 63.6% [30]. Based on our research results, which showed a 31.9% incidence rate of grade 2 and above RP, TRT can be safely treated in combination with bevacizumab.

There is no consensus on the mechanism by which bevacizumab use with TRT increases the incidence of RP, but some signal pathway may explain the relationship between bevacizumab and RP. On the one hand, the anti-VEGF receptor effect of bevacizumab can normalize tumour blood vessels, improve the hypoxic environment of tumour tissue and sensitize tumours to radiation. Anti-VEGF antibody inhibits the VEGF signalling pathways required for wound healing following typical tissue damage by radiation, suggesting caution in treating patients with combinations of targeted agents and radiotherapy. In preclinical studies, angiogenesis inhibitors have been shown to enhance radiation-induced cell killing and brief normalization of the tumour vascular system leading to increased oxygenation [31, 32]. Bevacizumab has also been shown to significantly downregulate the gene base and DNA repair involved in proliferation [33]. Whether the mechanism of tumour radiosensitization is also the reason for enhanced radiation-induced lung injury remains to be studied.

Based on the high-risk factors identified in our results, we provide some recommendations to reduce the incidence of RP. One of the high-risk factors for RP is radiation dosimetry, including V_20_ > 16.9% and PTV-volume > 183.85 cm^3^ is an independent predictor of RP occurrence. Therefore, it is necessary to strictly select the treatment population, and selecting smaller treatment targets may be a good suggestion for better patient protection. Also strictly limit the physical parameters of radiotherapy, balance the relationship between radiotherapy target area and radiation dose. At last, suspended the use of bevacizumab in a given cycle or increase the interval between bevacizumab and TRT is also a suggestion worth considering. It is worth mentioning that one patient adverse event occurred and the cause of death was massive haemoptysis caused by TEF in our study. Coincidentally, in a clinical study targeting NSCLC, 2 out of 5 patients developed TEF during TRT and bevacizumab maintenance treatment [34]. Two patients also had severe oesophageal toxicity after CRT and bevacizumab treatment. The implication is that the severe oesophageal toxicity caused by this treatment may make patients more susceptible to TEF. This adverse event is worth our vigilance.

In terms of survival, the combination of bevacizumab and TRT significantly prolonged the survival of patients in our study (mPFS, 14.27 months; mOS, 37.09 months) compared with the BEYOND study [2] (mPFS, 9.2 months; mOS, 24.3 months) and ECOG4599 [1] study (mPFS, 6.4 months; mOS, 12.3 months) reported the survival outcomes of first-line chemotherapy combined with bevacizumab (without TRT). The reason for this phenomenon may lie in the potential screening of the treatment population. Firstly, multiple previous studies have confirmed the significant effect of TRT on prolonging patient survival as mentioned in the background, and this is no exception in our study. Secondly, in our study, the majority of patients (89.1%) included TRT as consolidation therapy, which means these patients have a relatively good response to first-line treatment. In addition, the patients selected in our study were those who received first-line treatment, and patients who used bevacizumab after progression were excluded. This means that these patients have limited lesions and are relatively more likely to have a better prognosis. In summary, considering both the significant survival benefits obtained by patients and the acceptable incidence of RP, simultaneous bevacizumab and thoracic radiotherapy can be allowed. But it is necessary to require clinical doctors to do a good job of patient treatment follow-up and promote education on possible discomfort symptoms.

Several limitations should be acknowledged. Firstly, this was a retrospective study in a single institution, inevitably resulting in selection bias. Secondly, although we expanded the sample size as much as possible, an insufficient sample size is still a drawback. The reason for the small sample size may be that the previously reported high incidence of adverse reactions led clinical doctors to be more cautious when using bevacizumab and TRT. Effective intervention measures for RP are a direction of interest in subsequent research. The treatment for RP is only routine anti-inflammatory and hormone therapy, but some patients are still not treated. Therefore, there is an urgent need for an effective preventive drug to curb the occurrence of RP. A paper reported the protective effect of Erb-(IL-10)2, a bispecific protein synthesized by cetuximab and IL-10, on radiation-induced skin damage and mucosal repair, which may be helpful for the intervention of RP even oesophagitis radiation injury [35].

## Conclusion

In conclusion, the incidence of Grade 2 or above RP in combination with bevacizumab and TRT was 31.9%. V_20_ > 16.9% and PTV-volume > 183.85 cm^3^ were independent predictors of RP. The simultaneous use of bevacizumab and TRT is acceptable, but close follow-up and the occurrence of TEF should be noted.

## Data Availability

All data generated or analyzed during this study are included in this published article.

## Abbreviations

TRT: Thoracic radiotherapy
NSCLC: Non-small cell lung cancer
PFS: Progression-free survival
OS: Overall survival
RP: Radiation pneumonia
GTV: Gross tumor volume
PTV: Planning target volume
ADL: Activities of daily living
ROC: Receiver operating characteristic
CT: Computed tomography
PET-CT: Positron emission tomography/computed tomography
SBRT: Stereotactic body radiotherapy
TEF: Tracheoesophageal fistula
PD-L1/PD-1: Programmed cell death ligand-1/ programmed cell death-1
EGFR-TKI: Epidermal growth factor receptor tyrosine kinase inhibitor
VEGF: Vascular endothelial growth factor
